# Lung ultrasound in children with asthma exacerbation: a longitudinal study

**DOI:** 10.1007/s40477-025-01113-9

**Published:** 2026-01-31

**Authors:** Lucypaula Andrade Pinheiro Fernandes, José Dirceu Ribeiro, Mariana Beatriz Gomes de Abreu, José Dilbery Oliveira da Silva, Thaise de Abreu Brasileiro Sarmento, Silvia Inara Araujo Gomes

**Affiliations:** 1 Multicampi School of Medical Sciences of Rio Grande Do Norte, Federal University of Rio Grande Do Norte, Caicó, RN Brazil; 2 School of Medical Sciences, State University of Campinas (UNICAMP), Center for Research in Pediatrics (CIPED), Campinas, SP Brazil; 3 Federal University of Campina Grande, Campus Cajazeiras, Cajazeiras, PB Brazil; 4 Hospital Universitário Júlio Bandeira de Melo (EBSERH), Cajazeiras, PB Brazil

**Keywords:** Ultrasonography, Lung, Asthma, Child, Longitudinal studies

## Abstract

**Introduction:**

Lung ultrasound (LUS) is a non-invasive imaging method that does not use ionizing radiation, making it particularly useful for evaluating children and adolescents.

**Objectives:**

This study aimed to compare LUS findings in children and adolescents with asthma exacerbation to those with controlled asthma.

**Methods:**

A prospective longitudinal observational study was conducted, following the Strengthening the Reporting of Observational Studies in Epidemiology (STROBE) checklist for reporting observational studies.

**Results:**

Among patients with asthma exacerbation, 33 individuals were analyzed, and the study found that 51.5% of children in the exacerbation group had positive ultrasound findings, compared to only 12.1% in the controlled group. The calculated prevalence ratio was 2.27 (95% CI 1.46–3.55), with a *p*-value of 0.001, indicating a statistically significant difference in ultrasound findings between the two groups.

**Discussion:**

These findings suggest that lung ultrasound may be a useful tool for identifying changes in children with asthma exacerbations. The significantly higher prevalence of positive findings in the exacerbation group (51.5%) compared to the controlled group (12.1%) suggests that LUS has the potential to detect changes associated with asthma exacerbation. Further research, including multicenter studies, is needed to validate these findings.

**Conclusion:**

LUS demonstrated a higher prevalence of positive findings during asthma exacerbations, suggesting potential clinical utility as an adjunctive tool in pediatric asthma assessment.

## Introduction

### Context/rationale

Asthma is a disease typically characterized by chronic inflammation of the airways, which causes respiratory symptoms such as wheezing, shortness of breath, chest tightness, and cough, varying over time and in intensity [1] and it is the most common chronic disease in childhood [2]. Attacks are associated with variable airflow obstruction and inflammation in the lungs, which, if untreated, can be fatal, but with appropriate treatment, they can be reversible [3]. To provide the best possible care for patients with asthma, a detailed assessment should be conducted to accurately diagnose the condition and characterize its severity level [4].

Imaging evaluation of the chest has dramatically evolved over the past decade; however, the clinical diagnosis of asthma still relies on a compatible history, exam findings, and evidence of variable airflow obstruction. Chest imaging is more useful for assessing asthma complications and ruling out alternative diagnoses. Chest X-ray findings are non-specific and are often normal [5].

Recently, LUS has gained attention in pediatric respiratory assessment, due to its availability, reproducibility, low cost, and absence of ionizing radiation [6–12].

In asthmatic patients, there is still a lack of research directly comparing pulmonary ultrasound findings during asthma exacerbations with those obtained in controlled clinical states.

To our knowledge, no longitudinal studies have followed the same cohort of children during an acute exacerbation and later in a controlled phase to characterize changes detected by LUS over time. This gap limits understanding of whether ultrasound abnormalities are specifically linked to exacerbations or may persist even when symptoms improve.

This study addresses this gap by performing a longitudinal comparison within the same patients, aiming to identify LUS patterns associated with acute asthma exacerbations.

### Objectives

This study aims to evaluate lung ultrasound findings in children with asthma exacerbation and those with controlled asthma through a longitudinal study. The specific objectives were:

To determine the frequency of ultrasound changes in patients with asthma exacerbation compared to those with controlled asthma.

To identify the types of ultrasound changes, present at each time point (exacerbation and controlled asthma).

To assess the correlation between ultrasound findings and the severity of asthma exacerbation.

Predefined Hypotheses:

Patients with asthma exacerbation will have a higher prevalence of ultrasound changes compared to those with controlled asthma.

## Methods

This is a prospective longitudinal observational clinical study. The research required two evaluations: the first occurred during the patient’s hospitalization due to an asthma exacerbation, and the second, 1 to 2 years later, when the patient was in a controlled asthma state. The study was conducted in a general university hospital, responsible for the majority of pediatric care and hospitalizations. The study was approved by the Research Ethics Committee of the Federal University Hospital under approval number 4,650,667. Data collection took place between May 2022 and September 2024. Written informed consent was obtained from the parents or legal guardians at the first evaluation.

The selection occurred at the first time point, with the inclusion of children aged 2 to 14 years with an asthma exacerbation, excluding those with the first episode of dyspnea or other chronic respiratory or systemic diseases. The upper age limit was determined by the fact that 14 years is the upper age limit for admission to the institution. Children were identified and included in the cohort during the triage process when they presented with a second episode of dyspnea and wheezing, with no clinical findings suggestive of pneumonia. The diagnosis of asthma was presumed based on clinical symptoms, as the children did not undergo pulmonary function tests. Initially, 34 patients were included in the study. During the second evaluation, one patient did not meet the criteria due to a viral infection and was excluded, leaving 33 participants for the reassessment.

The asthma exacerbation was defined by the presence of wheezing, cough, and acute respiratory distress requiring hospitalization. The severity of the exacerbation was classified using the Pulmonary Respiratory Assessment Measure (PRAM) score, a validated instrument for grading asthma exacerbations, according to the PRAM Score Assessment for Asthma document from Child Health BC, which is publicly available on the official Child Health BC website. The PRAM categories were defined as mild (0–3 points), moderate (4–7 points), and severe (8–12 points). Hospitalization status was determined based on information recorded in the medical chart by the attending pediatrician at the time of the visit.

Asthma control was assessed using a specific questionnaire, and the second clinical evaluation was conducted, in most cases, by a pediatric resident. Pulmonary ultrasounds were performed by the same radiologist, following the same protocol at both evaluation points, assessing the anterior, posterior, and lateral surfaces of the chest. Pulmonary abnormalities were classified as positive (presence of consolidation, B lines, and pleural changes) or negative (presence of A lines and absence of positive findings). The pulmonary ultrasound was performed after the initiation of medication (oxygen and steroids), which was usually administered to the children at the time of hospitalization. The radiologist performing the lung ultrasound was blinded to the clinical severity classification of the children at the time of the examination.

Descriptive analyses were performed to summarize the data, with demographic characterization of nominal and numerical variables. Proportional comparisons were made to evaluate differences in results. To assess the statistical significance of the change in proportions of ultrasound findings, the McNemar test was used. The significance level adopted for the statistical tests was 5% (*p* < 0.05). All statistical analyses were performed using SPSS software, version 25 (IBM Corp., Armonk, NY, USA).

To mitigate sources of bias, structured questionnaires and validated tools were used to minimize information bias, and reporting bias was reduced by publishing all findings, both positive and negative.

## Results

During the asthma exacerbation, the average age of the participants was 5.52 ± 2.25 years. At the time of controlled asthma, the average age was 7.15 ± 2.35 years. Regarding gender, the proportion of boys and girls was the same at both time points: 25 (75.7%) boys and 8 (24.3%) girls. The severity of the asthma exacerbation was distributed as follows: 22 (66.7%) had mild asthma, 8 (24.2%) had moderate asthma, and 3 (9.1%) had severe asthma.

Among the patients with asthma exacerbation, a total of 33 individuals were analyzed, and 17 (51.5%) presented positive ultrasound findings, whereas in the controlled group, 4 (12.1%) showed positive findings (Table 1). This difference represents the primary comparison of interest in the study.

**Table 1:** Comparison of lung ultrasound findings between asthmatic patients in exacerbation and controlled asthmatic patients

		Exacerbation n (%)	Controlled n (%)	Prevalence Ratio	95% Confidence Interval	*p*-value*
Ultrasound Result	positive	17 (51.5%)	4 (12.1%)	2.27	1.46- 3.55	0.001
	negative	16 (48.5%)	29 (87.0%)			

*McNemar Test

The study results indicate a significant difference in lung ultrasound findings between children with asthma exacerbation and those in the controlled group. This demonstrates that patients in exacerbation are substantially more likely to exhibit detectable changes on ultrasound.

The prevalence ratio was 2.27, indicating that children with asthma exacerbation are more likely to show positive findings compared to those who were controlled. The 95% confidence interval (1.46–3.55) supports the strength of this association, and the *p*-value of 0.001 confirms statistical significance.

In the exacerbation group (Table 2), the most frequent findings were B-lines and small consolidations. In the controlled group, only B-lines were identified. In patients with exacerbation, as shown in Table 2, the types of changes included pleural effusion/irregularity in 2 patients (6%), B-lines in 16 patients (48%), and small consolidations (< 1 cm) in 12 patients (36%). In patients with controlled asthma, the types of changes included B-lines in 4 (12.1%) patients.

**Table 2:** Comparison of proportions of positive ultrasound findings

Type of finding	Asthma Exacerbation Patients (n = 33)	Controlled Asthma Patients (n = 33)
Consolidation n (%)	12 (36.0%)	0
B- Lines n (%)	16 (48.0%)	4 (12.1%)
Pleural Changes n (%)	2 (6.0%)	0

Table 3 presents the distribution of ultrasound findings according to exacerbation severity. The results indicate a higher prevalence of positive findings in patients with more severe exacerbations.

**Table 3:** Comparison of proportions of positive ultrasound findings and the severity of asthma exacerbation

	Pulmonary ultrasound findings	
Patients in asthma exacerbation	Positive	Negative
Mild n (%)	10 (43.4%)	12 (56.6%)
Moderate n (%)	4 (50.0%)	4 (50.0%)
Severe n (%)	3 (100.0%)	0

There was a clear increase in the frequency of positive findings with greater exacerbation severity, and all severe cases presented ultrasound abnormalities. In patients with mild exacerbation, positive findings were found in 36% of cases, while in moderate exacerbations, this value increased to 50%. In severe cases, 100% of patients presented positive findings.

## Discussion

In this study, we found that children with asthma exacerbation had a significantly higher prevalence of positive lung ultrasound findings (51.5%) compared to those in the controlled group (12.1%). The calculated prevalence ratio of 2.27 indicates that patients in exacerbation are more than twice as likely to present changes on ultrasound. This key result shows a clear association between clinical exacerbation and the presence of ultrasound abnormalities, supporting the role of lung ultrasound as a sensitive tool for detecting airway and pulmonary changes during acute asthma episodes.

There are already studies showing that pulmonary changes in patients with asthma exacerbation include consolidation, B-lines, and pleural changes [13–15] and that patients with asthma, even when not in an acute episode, can also present B-lines, consolidations, and pleural abnormalities on lung ultrasound [16]. However, until now, this is the first longitudinal study, allowing a direct comparison between asthma exacerbation and control conditions over time. The findings of lung ultrasound in patients during exacerbation in this study, as well as in previous studies [13–15, 17], were also observed in patients with wheezing and signs of respiratory infection, which raises the question of whether these pulmonary changes occur due to asthma or as a result of pulmonary involvement by respiratory infections, as observed in previous studies [18].

Considering the objectives of our study and its limitations, we interpret our results as evidence supporting the utility of lung ultrasound in the evaluation of children during asthma exacerbations. Our data are consistent with recent evidence [19, 20] showing that lung ultrasound provides valuable information for the clinical assessment of asthma severity and may help differentiate between pure asthmatic exacerbations and conditions with overlapping infectious features.

The results of this study indicated a higher prevalence of positive findings in patients with severe asthma exacerbation, suggesting that ultrasound may be a useful tool in identifying pulmonary changes in situations of greater disease severity. This gradient of findings across severity levels reinforces the potential role of lung ultrasound as a complementary marker of clinical worsening.

Among the limitations of the study, selection bias stands out, as the included patients may not accurately represent the broader population of children with asthma. However, since the inclusion and exclusion criteria were well-defined, this minimizes the risk and strengthens the internal validity of the results. Furthermore, the generalization of the findings is limited by the fact that the study was conducted in a specific clinical setting, which may restrict the applicability of the results to other healthcare contexts, especially due to the demographic variations of the patients and therapeutic approaches. To increase external validity, future research should be conducted in different settings with larger and more diverse samples.

Additionally, due to the limited sample size, the precision of the descriptive statistical estimates was restricted to one decimal place. This may affect the reliability of the means and proportions presented, making it necessary for future studies with larger samples to be conducted to validate these findings and improve the precision of the estimates.

Finally, the clinical implications of this study are relevant: lung ultrasound may serve as a rapid, radiation-free, bedside tool to support the assessment of pediatric asthma exacerbations, helping distinguish more severe cases and potentially reducing diagnostic uncertainty in emergency settings. Future studies should explore standardized ultrasound scoring systems, its use in treatment monitoring, and its integration into pediatric asthma management protocols.

## Conclusion

In this cohort, lung ultrasound detected pulmonary changes far more frequently during asthma exacerbations than during controlled asthma, supporting its use as a sensitive, radiation-free adjunct in pediatric assessment. The increase in positive findings with greater exacerbation severity further reinforces its clinical value. Future studies with larger and more diverse populations are needed to confirm these results and to define how LUS can be integrated into routine asthma care.

## Data Availability

The datasets generated and analyzed during the current study are not publicly available due to ethical and privacy restrictions involving pediatric patients but are available from the corresponding author on reasonable request.

## References

[1] Global Initiative for Asthma (GINA). Global strategy for asthma management and prevention. 2024.

[2] Asher MI, Rutter CE, Bissell K, Chiang CY, El Sony A, Ellwood E et al (2021) Worldwide trends in the burden of asthma symptoms in school-aged children: Global Asthma Network Phase I cross-sectional study. Lancet 398(10311):1569–1580. 10.1016/S0140-6736(21)01730-9 34755626 10.1016/S0140-6736(21)01450-1 PMC8573635

[3] National Institute for Health and Care Excellence (NICE). Asthma: diagnosis, monitoring and chronic asthma management. 2021. 32755129

[4] Corren J (2005) Evaluation and treatment of asthma: an overview. J Allergy Clin Immunol 115(4 Suppl):S725–S732

[5] Trivedi A, Hall C, Hoffman EA, Woods JC, Gierada DS, Castro M (2017) Using imaging as a biomarker for asthma. J Allergy Clin Immunol 139(1):1–10. 10.1016/j.jaci.2016.09.028 28065276 10.1016/j.jaci.2016.11.009 PMC5224930

[6] Iovine E, Nenna R, Bloise S, La Regina DP, Pepino D, Petrarca L et al (2021) Lung ultrasound: its findings and new applications in neonatology and pediatric diseases. Diagnostics (Basel) 11(4):633. 10.3390/diagnostics11040633 33916882 10.3390/diagnostics11040652 PMC8066390

[7] Joshi P, Vasishta A, Gupta M (2019) Ultrasound of the pediatric chest. Br J Radiol 92(1100):20190265. 10.1259/bjr.20190265 10.1259/bjr.20190058 PMC6724634 31095416

[8] Jaszczołt S, Polewczyk T, Dołęga-Kozierowska M, Woźniak M, Doniec Z (2018) Comparison of lung ultrasound and chest X-ray findings in children with bronchiolitis. J Ultrason 18(74):193–197. 10.15557/JoU.2018.0029 30427130 10.15557/JoU.2018.0029 PMC6442216

[9] de Souza TH, Nadal JAH, Peixoto AO, Pereira RM, Giatti MP, Soub ACS et al (2019) Lung ultrasound in children with pneumonia: inter-operator agreement on specific thoracic regions. Eur J Pediatr 178(9):1255–1260. 10.1007/s00431-019-03424-3 31312938 10.1007/s00431-019-03428-2

[10] Ionescu MD, Balgradean M, Filip C, Taras R, Capitanescu GM, Berghea F et al (2022) Lung ultrasonography for predicting the clinical outcome of complicated community-acquired pneumonia in hospitalized children. Med Ultrason 24(1):56–63. 10.11152/mu-3536 10.11152/mu-3124 34379711

[11] Ammirabile A, Buonsenso D, Di Mauro A (2021) Lung ultrasound in pediatrics and neonatology: an update. Healthcare (Basel) 9(8):993. 10.3390/healthcare9080993 34442152 10.3390/healthcare9081015 PMC8391473

[12] Musolino AM, Tomà P, De Rose C, Pitaro E, Boccuzzi E, De Santis R et al (2022) Ten years of pediatric lung ultrasound: a narrative review. Front Physiol 12:743622. 10.3389/fphys.2021.743622 10.3389/fphys.2021.721951 PMC8770918 35069230

[13] Dankoff S, Li P, Shapiro AJ, Varshney T, Dubrovsky AS (2017) Point-of-care lung ultrasound of children with acute asthma exacerbations in the pediatric ED. Am J Emerg Med 35(4):615–622. 10.1016/j.ajem.2016.12.007 28063721 10.1016/j.ajem.2016.12.057

[14] Varshney T, Mok E, Shapiro AJ, Li P, Dubrovsky AS (2016) Point-of-care lung ultrasound in young children with respiratory tract infections and wheeze. Emerg Med J 33(9):651–656. 10.1136/emermed-2015-204894 27107052 10.1136/emermed-2015-205302

[15] Marzook N, Gagnon F, Deragon A, Zielinski D, Shapiro AJ, Lands LC et al (2022) Lung ultrasound findings in asymptomatic children with asthma. Pediatr Pulmonol 57(10):2574–2579. 10.1002/ppul.25806 35794853 10.1002/ppul.26061

[16] Özkaya AK, Başkan Vuralkan F, Ardıç Ş (2019) Point-of-care lung ultrasound in children with non-cardiac respiratory distress or tachypnea. Am J Emerg Med 37(11):2102–2106. 10.1016/j.ajem.2019.01.018 31189496 10.1016/j.ajem.2019.05.063

[17] Attanasi M, Sferrazza Papa S, Porreca A, Sferrazza Papa GF, Di Filippo P, Piloni F et al (2023) Use of lung ultrasound in school-aged children with wheezing. Front Pediatr 10:1004317. 10.3389/fped.2022.1004317 10.3389/fped.2022.926252 PMC9869023 36699291

[18] De Rose C, Sferrazza MS, Verri P, Montemitro E, Buonsenso D, Bloise D et al (2022) Potential application of lung ultrasound in children with severe uncontrolled asthma: preliminary hypothesis based on a case series. Medicina (Kaunas) 58(2):141. 10.3390/medicina58020141 35200755 10.3390/medicines9020011 PMC8877587

[19] Hoffmann RM, Neuman MI, Du M, Monuteaux MC, Miller AF, Neal JT et al (2025) Lung ultrasound findings in children with asthma exacerbations. J Ultrasound Med 44(3):535–544. 10.1002/jum.16617 39565005 10.1002/jum.16617

[20] Topalušić I (2025) The usefulness of lung ultrasound in pediatric asthma: a comparative study. J Asthma. 10.1080/02770903.2025.2519096 40511909 10.1080/02770903.2025.2519096

